# Head rebound test in the clinical neurological examination of veterinary patients: a case example and discussion of Stewart and Holmes’ rebound phenomenon

**DOI:** 10.3389/fvets.2023.1180132

**Published:** 2023-05-24

**Authors:** Koen M. Santifort

**Affiliations:** ^1^IVC Evidensia Small Animal Referral Hospital Arnhem, Neurology, Arnhem, Netherlands; ^2^IVC Evidensia Small Animal Referral Hospital Hart van Brabant, Neurology, Waalwijk, Netherlands

**Keywords:** cerebellar disorders, spasticity, extension, clinical neurological examination, Holmes, Stewart, veterinary

## Abstract

In human medical neurology, the clinical neurological examination is variably augmented by specific tests that may be either unsuitable for veterinary patients or not included in the clinical evaluation of veterinary neurological patients due to clinicians presumably being unfamiliar with these tests. An example of the latter can be found in testing for the Stewart and Holmes’ rebound phenomenon (“rebound test”). In this article, a veterinary case example is presented in which a modified version of this test was performed (“head rebound test”). The interpretation of the results of this test is discussed, and the literature on the Stewart and Holmes’ rebound phenomenon and testing thereof is reviewed.

## 1. Introduction

Clinical veterinary neurologists are trained to perform neurological examination in a fairly standardized manner. Nevertheless, its exact contents may be variable depending on, among others, the clinician, species examined, patient cooperation, and the patient’s clinical status. In human medical neurology, the clinical neurological examination is variably augmented by specific tests that may be either unsuitable for veterinary patients or not included in the clinical evaluation of veterinary neurological patients due to clinicians presumably being unfamiliar with these tests. An example of the latter can be found in testing for the Stewart and Holmes’ rebound phenomenon (“rebound test”) (1–5). In veterinary literature, there is one textbook that mentions the term “head rebound phenomenon” in two chapters on the vestibular system in the context of bilateral peripheral vestibular disorders and cerebellar disorders, respectively (6):

“If the head and neck are extended, and then the support is suddenly withdrawn, the head may rapidly descend ventrally beyond the normal neutral position. This action is termed a head rebound phenomenon and is a clinical sign of cerebellar dysfunction but also may occur with bilateral peripheral vestibular dysfunction.”

In this report, the result of modified testing for this phenomenon in a dog is presented and discussed. The implications and use of this test (“head rebound test”) in the clinical neurological examination are discussed as well, based on human literature.

## 2. Example case and clinical application of the head rebound test

An 8-year-old female neutered Jack Russell terrier presented with acute onset of vestibulocerebellar signs. A 10-fold overdosage of enrofloxacin (50 mg/kg) was administered subcutaneously by another veterinarian the night before the presentation (the dog had been presented to that veterinarian for evaluation and treatment of diarrhea). A general examination revealed no abnormalities. Neurological examination revealed the following abnormalities: horizontal head excursions, wide-based stance, severe hypermetric (cerebellar) ataxia, hypertonic limbs and neck (spasticity), bilaterally decreased menace response, bilaterally absent vestibuloocular reflexes, vertical positional nystagmus (dorsal recumbency), clinically suspected deafness (no response to auditory stimuli), and a very prominent extension followed by overshooting flexion of the head and neck in response to forced flexion of the head and neck (“head rebound test”). The latter to the point of “catapulting” the front of the dog into the air. Supplementary Video S1 shows the performance and results of this clinical test. The test results were interpreted to be abnormal: the Stewart and Holmes’ rebound phenomenon was absent. Further diagnostic tests including hematology, biochemical blood tests, and high-field magnetic resonance imaging study of the head revealed no abnormal findings. An intoxication was presumptively diagnosed based on temporal relation to known marked overdosage of a fluoroquinolone antibiotic. Treatment consisted of metoclopramide 0.2 mg/kg *per os* q8 h for 2 days, omeprazole 1 mg/kg q12h, and assisted drinking and feeding. The dog was ambulatory and was able to drink and eat on its own by day 2. During follow-up over the next 2 months, the dog progressively improved and eventually recovered except for persistent hearing loss (suspected complete deafness), residual ataxia, and disequilibrium, which was most noticeable when the dog was observed standing up after sleeping or jumping from the couch.

## 3. Discussion

### 3.1. Stewart and Holmes’ rebound phenomenon in human medicine and the head rebound test in veterinary medicine

In human medicine, the “Stewart & Holmes’ rebound phenomenon” is a normal clinical finding in neurological examination. Its evaluation is of particular relevance in patients with spasticity and cerebellar disease (1–3, 7, 8). The term is applicable to the normal action of antagonistic muscles after a barrier is removed that inhibits the action of the flexor or extensor muscles. One way of testing for this phenomenon (involving spinal reflex pathways) is described here (note that flexion may be substituted for extension and vice versa) and based on literature descriptions in human medicine and descriptions and videos depicting the test online (1–3):^1^

With the patient in a standing or seated position, the patient is asked to extend one arm and flex at the elbow with the hand forming a fist upward (direction of the ceiling; 90 degrees flexion at the elbow). The examiner’s hand is placed against the palm of the hand and the patient is asked to withstand the force the examiner puts on the palm to attempt extension of the elbow, such that the arm remains in the same position. The examiner instructs the patient to keep the arm in that position. The sudden removal of the examiner’s hand results in some movement of variably normal extent in the direction of the force the patient was asked to provide (i.e., flexion of the elbow) which is reflexively countered by antagonistic muscles as the patient attempts to retain the tested arm in the desired position (reflex extension, “rebound”).

It is clear from this description that the Stewart and Holmes’ rebound phenomenon itself is not abnormal. Dr. Holmes indeed stated that the rebound phenomenon is present in non-affected (normal) limbs, exaggerated in spastic limbs (i.e., the action of antagonistic muscles is superfluous with exaggerated rebound), and absent in limbs affected by cerebellar disease (i.e., the action of agonistic muscles is superfluous and encountered with no rebound) (1–5). Thus, an exaggerated response or absence of rebound action of antagonistic muscles when testing for this rebound phenomenon is abnormal.^2^ The interpretation of the results of such a test is subjective as the extent of the rebound phenomenon varies between normal individuals. The results are usually classified as “normal” or “abnormal” according to the interpretation of the examiner. In the case of the latter, it is prudent to add “exaggerated” or “absent.” Methods to quantify the results of the test are reported as well (7, 8). Examples of different test methods for this phenomenon can be found on the internet. The reflexive nature of this phenomenon is evident though voluntary cooperation of the patient for the performance of this test is mandatory in usual clinical circumstances in human medicine.

The literature refers to this phenomenon or test with different terms, including “(no-) rebound phenomenon of Gordon Holmes,” “(no-) rebound phenomenon of Stewart and Holmes,” “Stewart–Holmes sign,” “‘Stewart–Holmes test,” “Holmes rebound test,” “Holmes rebound phenomenon,” or simply “rebound phenomenon” and “rebound test” (1–5, 7–11). Gordon Morgan Holmes and Thomas Grainger Stewart were human medical doctors and neurologists (although at that time possibly not named as such) that first described the phenomenon in the early 20th century, and it was Dr. Holmes that named it the “rebound phenomenon” (1, 2, 11). Some medical texts use these terms to imply abnormality of the described response (9, 10), while some (rightfully) describe abnormality pertaining to the findings when testing for this phenomenon (i.e., the phenomenon itself is not abnormal as previously explained). Some texts also describe the “sign” to be “positive” or “negative,” which can be confusing (12). The test is regarded as a measure of dyssynergia and results from a defect in the “orchestration of muscle synergies necessary to perform voluntary movements” (3, 7).

In one veterinary textbook that mentions the term “head rebound phenomenon,” the description includes an extension of the head and neck as opposed to flexion of the head and neck. Testing of the extensor or flexor muscles may be considered equivalent to the evaluation of the rebound phenomenon as in human medicine. The author proposes to use the term “head rebound test” in the clinical exam of veterinary patients and describe the response as either “normal” or “abnormal” and in the case of the latter as either “exaggerated” or “absent.” Interestingly, the inclusion of bilateral peripheral vestibular dysfunction in the veterinary textbook statement is not mirrored in the literature on the Stewart and Holmes’ rebound phenomenon in human literature, and the author was unable to find veterinary texts further explaining or describing its occurrence in bilateral peripheral vestibular disorders. Since human tests covering the Stewart and Holmes’rebound phenomenon usually involve testing of limbs (i.e., no mention of tests that pertain to the head are found) and the vestibular system is intimately involved in coordination and positioning of the head, this makes theoretical sense.

### 3.2. Case discussion

In the case reported here, both cerebellar and bilateral (peripheral) vestibular signs were present. Sensorineural (cochleosaccular) deafness was clinically suspected but not confirmed as brain stem auditory evoked potential testing was not performed. As such, the author cannot speculate on the exact neuroanatomical pathway dysfunction of the abnormal, exaggerated results of the head rebound test in this case. Although the temporal relationship to a known marked overdose of a fluoroquinolone antibiotic was highly suggestive and a toxic etiology would fit with the recovery of most of the clinical signs with only supportive treatment, there are no reports in the literature on fluoroquinolone intoxication in dogs resulting in auditory loss and cerebellovestibular signs. The product information of injectable enrofloxacin products for veterinary use lists neurological disorders as a possible side effect though this is not specified, and there are no documented effects of an overdose in dogs. Neurological and other adverse effects of fluoroquinolones are reported in human beings as well as in veterinary medical literature (horses and cats). These include but are not limited to “hearing, vestibular and special senses disorders” (including deafness), ataxia, nystagmus, (epileptic) seizures, and blindness (13–21). Enrofloxacin is uncommonly used in human medicine (22), and adverse effects in dogs like the reported signs in the case reported here are undocumented (21). The main metabolite of enrofloxacin in dogs is ciprofloxacin (23). This fluoroquinolone has been linked to ototoxicity in humans and experimental animal models though late topical therapy in recommended doses for otitis with this drug is considered to be safe (24, 25). Enrofloxacin or ciprofloxacin are not specifically reported to be ototoxic in dogs (26). In a recent study, 20 mg/kg enrofloxacin administration to prairie dogs was considered to be safe, and no adverse effects were documented (27).

The head rebound test and the results in this example case correlate with the presence of cerebellar dysfunction. This exemplifies the possible value of performing the head rebound test in veterinary neurological patients. Regarding the interpretation of the head rebound test in the case reported here, it was interpreted as abnormal and absent. At first glance, the reader could possibly interpret the video images to show an exaggerated response. This is not the case as the rebound phenomenon pertains to the action of antagonistic muscles (in this case, flexor muscles of the head and neck). There is no rebound as such but rather an overextension to the limit. This is a sign of head and neck hypermetria or dyssynergia of head and neck muscles, suggestive of cerebellar dysfunction in accordance with Dr. Holmes’ interpretations or possibly bilateral (peripheral) vestibular dysfunction as discussed above.

## 4. Conclusion

The use of non-standard tests in the clinical neurological examination of veterinary patients such as the head rebound test may be of use for clinical neuroanatomical localization. The use of this specific test in cases with cerebellar and bilateral (peripheral) vestibular dysfunction can provide more information on its usefulness and the interpretation of the results of this test in veterinary patients.

## Data availability statement

The original contributions presented in the study are included in the article/Supplementary material, further inquiries can be directed to the corresponding author.

## Ethics statement

Ethical review and approval was not required for this case report since the animals were treated according to the local legislation and institutional requirements. Written informed consent was obtained from the owners for the participation of their animal in this study and for the publication of any potentially identifiable images/videos or data included in this article.

## Author contributions

The author confirms being the sole contributor of this work and has approved it for publication.

## Funding

The publication fee was covered by IVC Evidensia’s fund for publication of peer-reviewed scientific articles.

## Conflict of interest

The author declares that the research was conducted in the absence of any commercial or financial relationships that could be construed as a potential conflict of interest.

## Publisher’s note

All claims expressed in this article are solely those of the authors and do not necessarily represent those of their affiliated organizations, or those of the publisher, the editors and the reviewers. Any product that may be evaluated in this article, or claim that may be made by its manufacturer, is not guaranteed or endorsed by the publisher.

## Footnotes

^1^Example 1: https://www.youtube.com/watch?v=nEH6ROgeulE, 2: https://www.youtube.com/watch?v=alGXuF1aB14, 3: https://www.youtube.com/watch?v=_SiZLBQPHGk and 4: https://stanfordmedicine25.stanford.edu/the25/cerebellar.html.

^2^When performing the test in human beings, clinicians are warned to take precautionary measures to prevent the patient from hitting him−/herself in case of an absent rebound phenomenon suggestive of cerebellar disorders. Note the padding of the floor of the cage in the video of the dog to prevent injury due to overshooting flexion of the head and neck after correction for the absence of the rebound phenomenon (see the section Discussion on interpretation of the head rebound test in this case).

## Supplementary material

SUPPLEMENTARY VIDEO S1 The head rebound test in the dog of the described case example.
